# Assessing Climate Impact: Distribution Modeling and Conservation Assessments of *Sesamum* (Pedaliaceae) Species

**DOI:** 10.1002/ece3.71387

**Published:** 2025-05-13

**Authors:** Daniel A. Zhigila, Nawal Shrestha, Zainab A. Abubakar, A. Muthama Muasya

**Affiliations:** ^1^ Department of Plant Science Gombe State University Tudun Wada Gombe State Nigeria; ^2^ The Bolus Herbarium, Department of Biological Sciences University of Cape Town Cape Town South Africa; ^3^ Department of Organismic and Evolutionary Biology Harvard University Herbaria Cambridge Massachusetts USA

**Keywords:** climate change, extinction risk, niche breadths, phylogenetic signal sesamum, species distribution modeling

## Abstract

Plants with restricted distributions and small population sizes are particularly vulnerable to climate change. *Sesamum* species are ideal for species distribution modeling due to their ecological sensitivity, agricultural and economic importance, and wide geographic range, providing insights for conservation and policy. We applied the maximum entropy (MaxEnt) model to assess the global ecological niche breadth of *Sesamum* species and examine how bioclimatic and soil variables influence their future (2080) distribution. We identified key environmental drivers and projected species‐specific range shifts under changing climatic conditions. MaxEnt models effectively predicted suitable habitats, with climate variables playing a dominant role. Precipitation of the wettest month (BIO13) was particularly influential for *S. abbreviatum, S. alatum*, and *S. angustifolium* , while temperature variables (BIO7, BIO11) were also key. Elevation moderately impacted *S. angolense* , while soil factors such as pH ( *S. abbreviatum* ) and clay content ( *S. angolense* ) exhibited species‐specific effects. Principal component analysis revealed variation in niche breadth, with *S. indicum* and *S. schinzianum* occupying broader ecological ranges, whereas *S. saxicola* and *S. abbreviatum* were more restricted. Future projections suggest 46.4% of the species will experience range contractions, with *S. schinzianum* facing the most significant decline. Conversely, 39.3% of the species, including *S. imperatricis* and *S. abbreviatum* , are expected to expand their ranges. Phylogenetic analyses indicate a random distribution of niche breadth and extinction risk across the genus. Our findings highlight the susceptibility of *Sesamum* species to climate change, emphasizing the need for urgent conservation actions. Prioritizing vulnerable species such as *S. forbesii* and *S. sesamoides*, alongside habitat restoration and long‐term monitoring, is crucial to mitigate population declines and prevent extinction.

## Graphical abstract

Sesamum species, known for their sensitivity to environmental conditions and narrow distribution ranges, were modeled using MaxEnt to predict how climate change will impact their future habitats. The study found that 74% of the species are expected to experience reduced niche breadths and range contractions by 2080, while a few species may expand their ranges. Precipitation and temperature were key drivers of distribution, emphasizing the need for targeted conservation efforts for vulnerable species like *S. forbesii* and S. sesamoides.

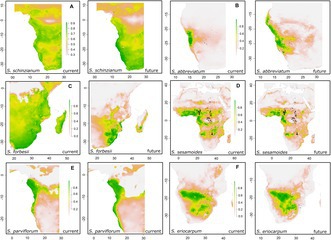

## 1 Introduction

The genus *Sesamum* L., comprising approximately 31 accepted species, is the largest in the Pedaliaceae family (Bedigian 2015; APG IV 2016; POWO 2025). It is native to tropical and subtropical regions of Africa, Asia, and Australia, with Africa hosting the greatest species diversity. Sub‐Saharan Africa harbors 28 species, while the Indian subcontinent has three species, supporting the hypothesis of an African origin (POWO 2025; Zhigila and Muasya 2023). The origin and biogeography of *Sesamum* have been subjects of debate (Gormley et al. 2015; Zhigila and Muasya 2023). Early hypotheses suggested that *Sesamum* originated in tropical Africa and spread to other regions, including the Middle East and the Indian subcontinent (Kobayashi 1986; Bedigian 2004). More recent studies support a vicariance‐based origin from southern Africa, with subsequent diversification into India and Australia (Zhigila and Muasya 2023). Several *Sesamum* species are cultivated for their seeds, which are valued for their high oil content. For example, *Sesamum indicum* L., the type species, is widely cultivated across Africa, Asia, the Americas, and Europe (Bruce 1953; Bedigian 2003, 2015; Zhigila et al. 2015). Given its wide global distribution, values, and presence in biodiversity hotspots, *Sesamum* provides an excellent model for studying the impact of climate change on tropical and subtropical flora.

Species distribution modeling (SDM) is a widely used approach that integrates ecology, geography, and data science to estimate species' ecological niches and predict potential range shifts due to climate change (Elith and Leathwick 2009; Urbina‐Cardona et al. 2019). SDMs have proven valuable in assessing species' vulnerability to extinction, especially in the context of rapidly changing environmental conditions (Condamine et al. 2013; Yessoufou and Davies 2016). These models, which incorporate topographical and climatic data, provide critical insights into species' geographical ranges, aiding in biodiversity conservation and policy decisions (Kier and Barthlott 2001; Franklin 2023).

Understanding the distribution patterns of *Sesamum* species, particularly in relation to climate change, is crucial for several reasons: (i) *Sesamum* species have significant agricultural and economic value, and predicting their distribution shifts is essential for food security and optimizing agricultural practices (Ashri 1998); (ii) their sensitivity to environmental conditions and genetic diversity make them good indicators for assessing climate change impacts (Laurentin 2009; Franklin 2023); (iii) the geographic range of *Sesamum* species spans regions expected to experience significant climatic shifts (Bedigian 2010; Zhigila and Muasya 2023); and (iv) SDM insights can inform conservation and adaptation strategies on local, regional, and global scales (Elith and Leathwick 2009; Floury et al. 2021).

The interaction between environmental factors and evolutionary history shapes species' distributions over time and space (Münkemüller et al. 2015; Franklin 2023). Interestingly, the signature of past evolutionary processes is commonly obtained on phylogenetic trees. These phylogenies can be used to account for the relatedness of species in biogeographic, comparative, and evolutionary studies (Lavergne et al. 2010; Münkemüller et al. 2015). Phylogenetic analyses provide insights into species' evolutionary relationships and can be used to examine extinction risks and conservation priorities (Davies et al. 2011). Testing the phylogenetic signal of factors such as extinction risk can reveal whether species face threats due to shared evolutionary histories or independent ecological factors (Yessoufou and Davies 2016). Phylogenetically structured extinction risks suggest that certain lineages may be more vulnerable, highlighting functional traits critical to understanding species' survival (Kelly et al. 2014; Ferraz et al. 2021; Zhigila et al. 2023).

Advances in high‐resolution environmental data (Hijmans 2017; Karger et al. 2017; Vieilledent et al. 2018) have enhanced the ability to model species' niche breadths (Lannuzel et al. 2021). This study uses maximum entropy (MaxEnt) modeling to: (i) construct SDMs for *Sesamum* species, predicting their current and future distribution patterns based on climate, soil, and elevation variables; (ii) identify key environmental correlates influencing species distributions; (iii) assess the potential for range shifts in response to climate change; (iv) explore the relationship between species niche breadth and range size; and (v) test whether extinction risks in *Sesamum* are phylogenetically structured.

## 2 Methods

### 2.1 Species Occurrence Data

**FIGURE 1:**
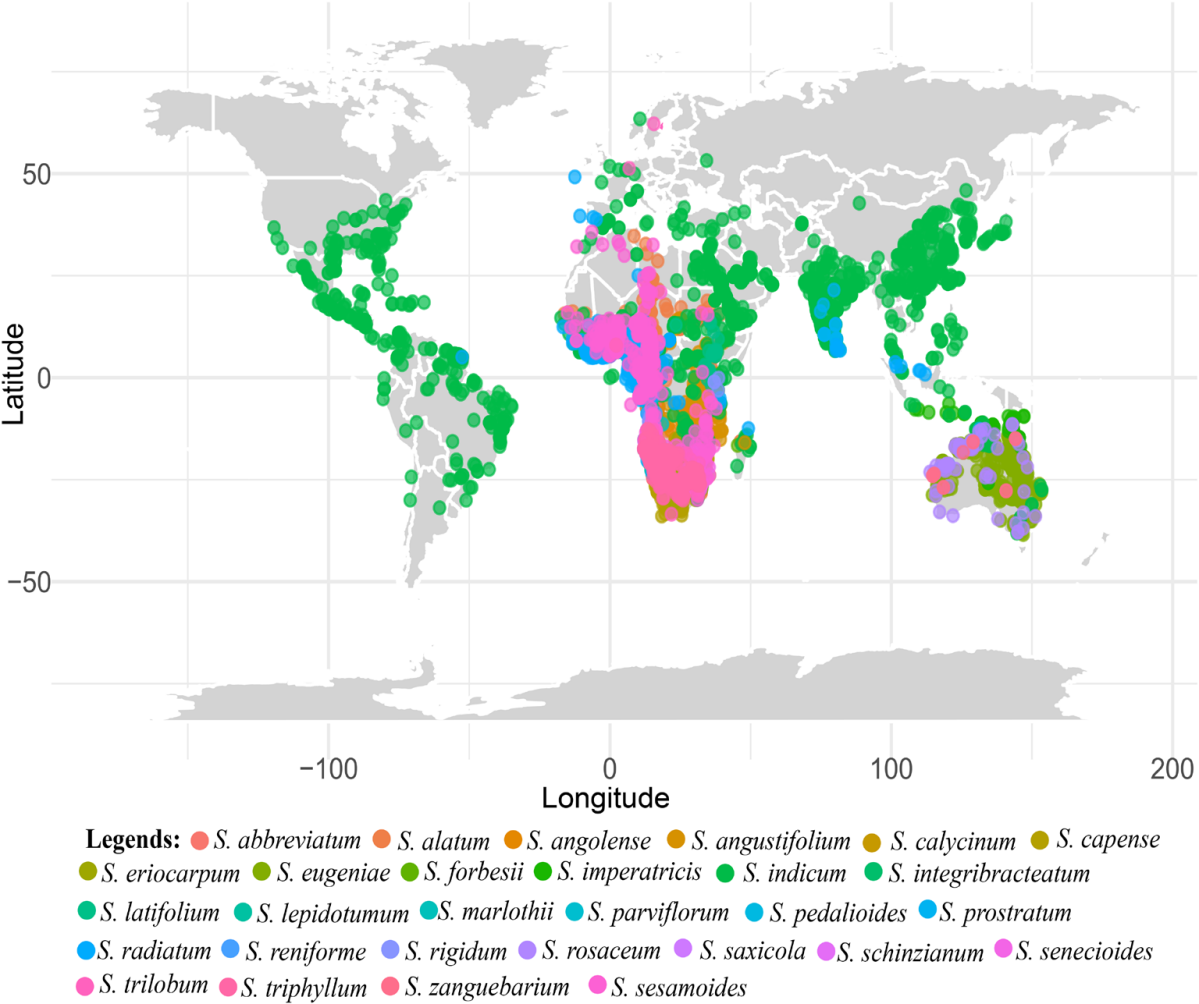
Global distribution of *Sesamum*.

We generated a database of *Sesamum* occurrence points throughout their distribution range (Figure 1; Table S1) using different sources, including species data at the Global Biodiversity Information Facility websites GBIF (2024) GBIF.org Occurrence Download https://doi.org/10.15468/dl.ja9jv3, published manuscripts, citizen science data points (iNaturalists 2025), and specimen locality information on herbarium labels at national and international herbaria holding significant *Sesamum* materials, e.g., ABUH, BOL, FHI, GSUH, K, MO, OFX, and S codes following (Thiers 2025) and our field collections. The locality information was used to obtain coordinates by geo‐referencing all specimens. At the geo‐referencing stage, each specimen's coordinates were given a precision code, making it possible to select specimen subsets based on locality precision. We used ArcMap GIS 10.6.1 (2021) for spatial filtering to remove duplicate and erroneous records, ensuring data consistency. Specifically, we applied a spatial thinning approach to reduce sampling bias and removed records outside the known ecological range of *Sesamum* species.

All specimens were appropriately checked against the species types on JSTOR (2025), POWO (2025) or herbaria to avoid misidentifications. The model excludes species with fewer than 10 point‐occurrence records since several studies (e.g., van Proosdij et al. 2016) have proven that small sample numbers impair SDM accuracy. As a result, only species with 10 or more occurrence data were considered for further investigation. We also excluded specimens with multiple entries (duplicates) from a single locality or duplicate specimens from the same collector deposited in several databases. Finally, 6186 specimens representing 29 (of the 31 spp) *Sesamum* species were chosen from the initial database of 9910 species records (Table S1).

### 2.2 Predictor Variables

We used bioclimatic variables as environmental predictors obtained from the georeferenced current species locality points to assess the environmental niche of each species. The environmental predictors were queried within a GIS framework using raster package version 23.6‐30 (Hijmans 2024) in r (R Core Team 2024). Climatic layers were downloaded from Worldclim v. 2.1 (2022) at 30″ arc spatial resolution (fine scale with 2.5 × 2.5 km grid cells). We included the means and standard deviations of the annual temperature and rainfall records for the last 50 years until the year 2000, representing the present‐day climate scenario (Hijmans 2017; Hijmans et al. 2023); and the emission scenario projected by the Intergovernmental Panel on Climate Change (IPCC) of high representative concentration pathways (RCP 8.5) emissions for the year 2080 (Worldclim 2022). For elevation data, we used Shuttle Radar Topographic Mission elevation dataset (NASA 2020). Soil characteristics play a crucial role in determining plant niches (Jiang et al. 2022). Therefore, along with climatic conditions and elevation, we incorporated soil properties obtained from SoilGrids 250 m (https://soilgrids.org) at 15–30 cm depth. We assumed soil variables to remain unchanged under climate change.

To avoid compromise in model precision and overfitting as suggested by Feng et al. (2019), multicollinearity tests using pairwise Pearson correlation coefficients were performed (Pearson et al. 2006; Dormann et al. 2013). Hence, only one of the highly correlated variables with appropriate thresholds of *r* ≥ 0.75 (Dormann et al. 2013) was systematically retained for the downstream analyses. Finally, 15 noncollinear variables were employed for ecological niche modeling, comprising eight climatic variables, namely bio2 = Mean Diurnal Range (°C), bio5 = Max Temperature of Warmest Month (°C), bio7 = Temperature Annual Range (°C), bio11 = Mean Temperature of Coldest Quarter (°C), bio13 = Precipitation of Wettest Month (mm), bio14 = Precipitation of Driest Month (mm), bio15 = Precipitation Seasonality (mm), bio19 = Precipitation of Coldest Quarter (mm); four soil data, clay (%, w/w), nitrogen (%, w/w), pH (acidity or alkalinity, pH_KCl_) and organic carbon (%, w/w) and elevation (m).

### 2.3 Species‐Specific Estimation of Ecological Niche Breadth

The ecological niche breadth of each species was quantified by calculating the standard deviation for each environmental variable across all its current locality points. These factors were then summarized using a principal component analysis (PCA) to generate an overall index of niche breadth as described by Júnior and Nobrega (2018). Species‐specific ecological niche breadth was quantified by using a PCA of environmental variables in the r function prcomp (R Core team 2024). For each species, we z‐standardized environmental variables and calculated the standard deviation of each variable across its sampling points. We ran a PCA on these data to assess the differences in ecological niche breadth between species. The PC scores here represent the comparative niche breadths within the genus. The resulting ordination was employed to test the hypothesis that species with a narrow ecological niche breadth would exhibit a lower standard deviation relative to the mean, while species with a broad distribution were expected to show higher values (Gaston and Blackburn 2000; Zhang et al. 2023; Huang et al. 2024). To explicitly link our interpretation of PC1 as a concept of niche breadth and to established ecological niche overlap theory (MacArthur 1972), we quantified the relationship between PC1 and range size (log‐transformed) for both present and future using Pearson correlations. This provided statistical support for our claim that higher PC1 scores are associated with larger range sizes.

### 2.4 Species Distribution Modeling

We used the MaxEnt model (Phillips et al. 2006; Elith et al. 2011) as implemented in the dismo package (Hijmans et al. 2023) to model suitable habitats for *Sesamum*. The MaxEnt model is the most suitable package used for SDMs (Phillips et al. 2017; Soberón et al. 2017; Urbina‐Cardona et al. 2019; Ahmadi et al. 2023) and was developed in the context of chosen contemporary environmental layers. We ran species‐specific MaxEnt model runs; for each species, we adjusted the study area to include a 10° latitude and longitude buffer around the sampling points, as this was a reasonable estimation of the potential area of expansion possible due to climate change. To test the hypothesis that rapidly increasing climate change (Peterson et al. 2012; Morueta‐Holme et al. 2013) will impact species niche breadth for *Sesamum*, we ran separate Maxent models for both the current and future environmental climatic variables, with only the soil and elevation predictors kept constant. By comparing species' projected ranges at that time with their contemporary ranges, it was possible to quantify, for each species, the absolute area of climatically suitable habitats remaining in 2080, the percentage of range loss or expansion, the measure between the contemporary and projected ranges, and the extent to which species ranges differed.

For generating the models, 75% of each species' occurrence data was used to train the algorithm and the remaining 25% was used as test data, with the default 1000 background points used. The model quality was evaluated using the area under the receiver operating characteristic (ROC) curve (AUC) values. An AUC score of < 0.8 was considered random, from 0.8 to 0.9 was good, and > 0.9 was excellent (Fielding and Bell 1997). Thereafter, using the models thus derived, species suitable ranges were predicted.

### 2.5 Assessing Phylogenetic Signal

The recent phylogenetic tree developed by Zhigila and Muasya (2023) was adopted for testing phylogenetic signals. Briefly, Zhigila and Muasya (2023) generated a Maximum Clade Credibility in BEAST2 version 2.5.2 (Bouckaert et al. 2019). For the molecular evolution model, the GTR + I + Γ model was selected for both partitions using ModelTest‐NG (Darriba et al. 2019). Then, time calibration and construction of BEAST‐derived chronograms were conducted for inferring ancestral characters of species in *Sesamum*. The resulting chronogram was imported into R, where outgroup species and duplicate accessions were trimmed to match the spatial data matrix species. To determine whether range size (PC1 values) and extinction risk (change in range size) are phylogenetically structured (e.g., if species at greatest risk are expected to cluster within a clade), Blomberg's K (Blomberg and Garland 2002) and Pagel's λ (Pagel 1999) models were applied. These models assess the phylogenetic signal's strength, indicating whether species with higher extinction risks are clustered phylogenetically (Thuiller et al. 2011). Under the assumption of random Brownian motion, the alternative hypothesis that Blomberg's K and Pagel's λ are < 1.0 was tested against the null hypothesis that *K* and λ statistics are ≥ 1.0. A random pattern in the data would support the null hypothesis, so only *K* or λ values with significant *p*‐values (*p* < 0.05) were interpreted as structured patterns, indicating a strong phylogenetic signal. For both *K* and λ, 999 randomizations were used to compute *p*‐values. The phylosignal functions from the picante (Kembel et al. 2010) and geiger (Harmon et al. 2019; Keck et al. 2019) packages in R were utilized for these statistics.

## 3 Results

### 3.1 Model Evaluation and Variable Contributions

The Maxent model shows high predictive performance for *Sesamum*, with different environmental variables contributing to the habitat suitability for each species (Table 1; Table S1). The ROC (AUC) values are generally high (with an average score of 0.92, close to 1; Table 1), indicating that the Maxent model is effective in predicting the suitable habitats for these species. The habitat suitability of Sesamum species is influenced by a combination of elevation, soil properties, and climatic variables, with varying contributions across species (Table 1). Climate variables played a dominant role, particularly precipitation‐related factors such as precipitation of the wettest month (BIO13), which contributed significantly to the models of *S. abbreviatum* (19.46%), *S. alatum* (37.29%), and *S. angustifolium* (31.31%). Temperature‐related variables, including temperature annual range (BIO7) and mean temperature of the coldest quarter (BIO11), also had notable contributions, particularly in *S. angustifolium* (24.86%) and *S. alatum* (20.41%), respectively. Elevation had a moderate effect on species distributions, with its highest contribution observed in *S. angolense* (17.60%) but negligible influence on *S. abbreviatum* . Soil factors, though less influential than climate, contributed substantially in some cases, particularly soil pH in *S. abbreviatum* (22.87%) and clay content in *S. angolense* (16.03%). Despite a continuum in the influence of climatic versus soil variables on distribution, the environmental variables contributing to the models vary significantly between species. Although each species has unique ecological requirements and responds differently to various climatic and edaphic factors, these findings highlight the significant role of climate in shaping the distribution of *Sesamum* species, while soil and elevation exert species‐specific influences.

**TABLE 1:** *Sesamum* species and their corresponding ROC (receiver operating characteristic) AUC (area under the curve) values along with the percentage contributions of different environmental variables.

Species	AUC	Elev.	Soil contribution				Climate contribution							
		elev.	clay	N	pH	SOC	BIO2	BIO5	BIO7	BIO11	BIO13	BIO14	BIO15	BIO19
*S. abbreviatum*	0.984	0	16.028	7.03	22.867	1.142	13.883	2.087	7.697	0	19.458	2.475	7.189	0.144
*S. alatum*	0.867	6.752	5.965	3.101	2.544	1.932	1.518	7.987	3.112	20.407	37.287	3.657	2.961	2.776
*S. angolense*	0.926	17.602	3.044	0.192	16.443	4.245	15.076	17.951	2.496	1.579	11.6	3.407	0.469	5.896
*S. angustifolium*	0.914	3.671	2.942	1.17	16.603	1.039	2.746	2.409	24.859	3.614	31.313	1.37	2.505	5.759
*S. calycinum*	0.907	0	11.127	0.87	0	1.908	0	68.205	17.843	0	0	0	0	0.048
*S. capense*	0.929	1.387	1.924	0.876	8.72	1.465	1.561	1.289	4.871	4.483	64.572	1.98	4.973	1.9
*S. eriocarpum*	0.897	5.945	1.649	0.293	8.068	3.545	0.238	5.3	52.968	2.276	7.319	1.806	8.298	2.297
*S. eugeniae*	0.801	9.126	16.834	1.07	1.825	4.154	4.016	1.735	34.759	3.846	0.526	8.351	11.243	2.515
*S. forbesii*	0.866	4.042	6.243	0.663	7.324	7.165	0	11.962	0	0.035	0	7.411	5.3	49.855
*S. imperatricis*	0.98	0.21	0	0.001	0.735	3.939	65.256	0	0.531	1.23	0	11.713	9.745	6.64
*S. indicum*	0.845	11.692	0.936	3.106	9.093	0.852	8.889	6.799	1.35	37.132	10.742	2.573	4.673	2.163
*S. integribracteatum*	0.921	1.518	0.066	4.008	0.225	2.604	43.003	0.642	2.498	0.184	3.125	22.336	0	19.792
*S. latifolium*	0.986	0.77	21.883	0.258	25.107	0.037	2.043	15.057	1.041	6.239	1.967	3.511	0	22.089
*S. lepidotum*	0.962	4.113	0	2.04	29.091	2.481	0.982	0.464	0.198	0	0.132	1.733	16.921	41.845
*S. marlothii*	0.968	1.765	0.682	0.245	27.314	0.131	0.703	1.175	9.175	0	8.323	0.151	28.967	21.37
*S. parviflorum*	0.905	0	0	0	0	0.389	54.472	0.374	0	0	0	1.805	0	42.961
*S. pedalioides*	0.954	0.071	0	7.531	0	0	4.422	0	36.305	0	21.149	29.2	0	1.322
*S. prostratum*	0.885	9.42	0	0	0	0	0	0.533	0	45.29	0.403	39.25	0	5.104
*S. radiatum*	0.944	0.32	6.865	0.82	4.659	0.961	4.079	0.269	10.068	21.914	17.524	6.417	7.359	18.745
*S. reniforme*	0.908	1.746	0	12.62	0.811	0	0	11.4	20.88	0	2.457	42.907	0	7.179
*S. rigidum*	0.979	0.799	1.597	0.45	13.154	1.148	7.071	1.113	4.607	2.778	10.756	23.001	5.362	28.164
*S. rosaceum*	0.898	1.065	10.236	10.84	3.296	4.835	2.052	16.592	2.614	1.247	1.639	34.219	10.637	0.724
*S. saxicola*	0.999	32.846	0	0	0.767	0.22	0	0	1.22	4.887	15.108	7.234	1.424	36.294
*S. schinzianum*	0.71	0	0	0	0	0	0	61.417	0	0	0	38.583	0	0
*S. senecioides*	0.969	1.787	1.033	0.113	2.739	0.643	0.352	1.523	10.245	24.424	5.664	34.168	15.763	1.549
*S. sesamoides*	0.895	0.86	9.461	2.918	4.438	2.666	0.599	1.637	4.456	1.453	54.351	1.203	8.598	7.36
*S. trilobum*	0.958	0.667	0.334	4.939	2.4	0	0.593	23.14	21.687	9.286	15.178	5.182	4.278	12.316
*S. triphyllum*	0.9	0.164	1.414	0.026	25.062	24.36	0.528	3.901	1.842	18.088	10.996	1.104	12.49	0.027
*S. zanguebarium*	0.899	0	0	0	0	0	10.209	52.28	0	0	0	37.511	0	0
**Average**	**0.916**													

### 3.2 Index of Niche Breadth

**FIGURE 2:**
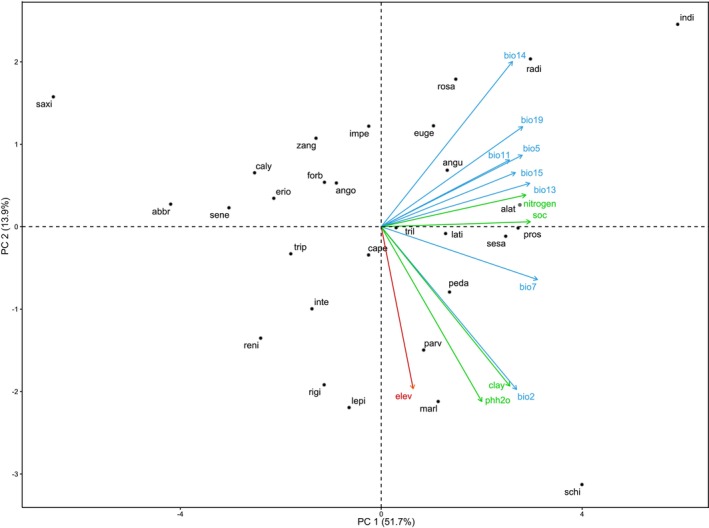
Principal component analysis (PCA) plot of the *Sesamum* species‐specific deviations from the original means of environmental correlates. Each black dot represents an ordination score for individual *Sesamum* species. The position of each dot in the plot shows how that species deviates from the mean environmental conditions (e.g., climate (blue), elevation (crimson), and soil (green)). The PCA plot helps to visualize and interpret how different environmental factors (climatic, elevation, soil) influence the distribution and variance among *Sesamum* species. The orientation and length of the vectors, along with the distribution of the species' scores, provide insights into the environmental preferences and ecological niches of these species.

The PCA plot of the *Sesamum* species‐specific deviations from the original means of environmental correlates provides a visual representation of how different *Sesamum* species are influenced by various environmental factors (Figure 2). PC1 captures the largest amount of variance (51.7%) while PC2 captures the second largest amount (13.9%) and together accounts for 65.6% of the total variability. Here, PC1 represents the species' niche breadths, indicating how broadly or narrowly a species can utilize its habitat. Higher PC1 (index of species niche breadth) scores suggest a broader niche breadth, implying that the species with the highest PC1 show large range size and species with low PC1 indicate small range size. For example, *S. indicum* and *S. schinzianum*, with higher PC1 scores of 5.5 and 4.0, respectively, have broader niche breadths. In other words, they can thrive in a wider range of habitats. Conversely, *S. saxicola* and *S. abbreviatum* , with −6.5 and −4.2 PC1 values, are restricted to smaller range breadths. Supporting the contribution of each environmental factor, mean diurnal range temperature and precipitation positively contribute to PC1 (0.5), suggesting that species thriving in a range of temperature and precipitation conditions have broader niches. Soil type negatively contributes to PC1, indicating that species with specific soil requirements, for example, *S. trifolium* , have narrower niches (Figure 2).

**FIGURE 3:**
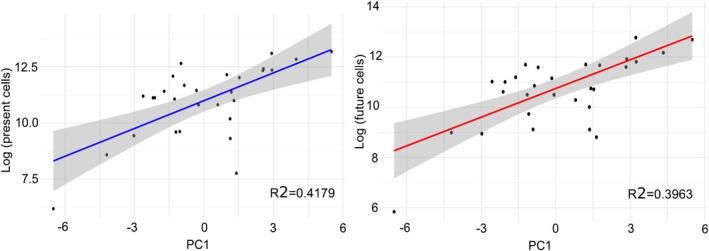
A correlation analysis quantifying the relationship between PC1 and range size supporting the claim that higher PC1 scores are associated with larger range size.

The correlation analysis shows a significant positive relationship between PC1 and log‐transformed cell sizes (Figure 3), both at present and in the future (*p* < 0.001). The models explain 41.79% and 47.75% of the variance in cell sizes, respectively, indicating a moderate fit. *F*‐statistics confirm the overall significance of both models, and residuals are symmetrically distributed with no extreme outliers, suggesting a good fit of the models to the data. The intercept indicates that when PC1 is zero, the predicted log‐transformed number of cells is approximately 10.75 (Figure 3).

### 3.3 Current and Potential Future Distribution Pattern

**FIGURE 4:**
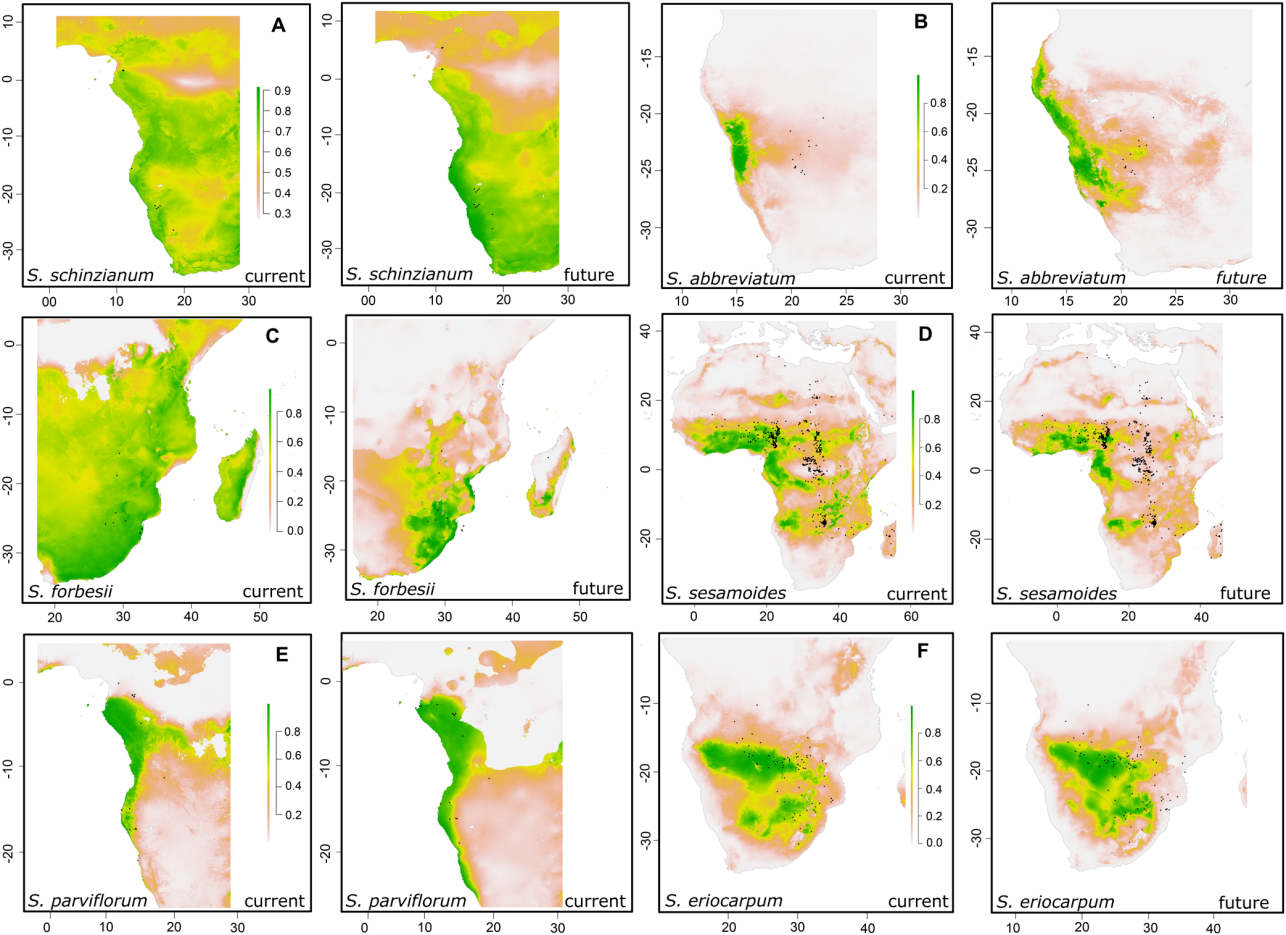
Comparative habitat suitability and niche breadths of exemplary *Sesamum* species based on the present and future predictions.

**FIGURE 5:**
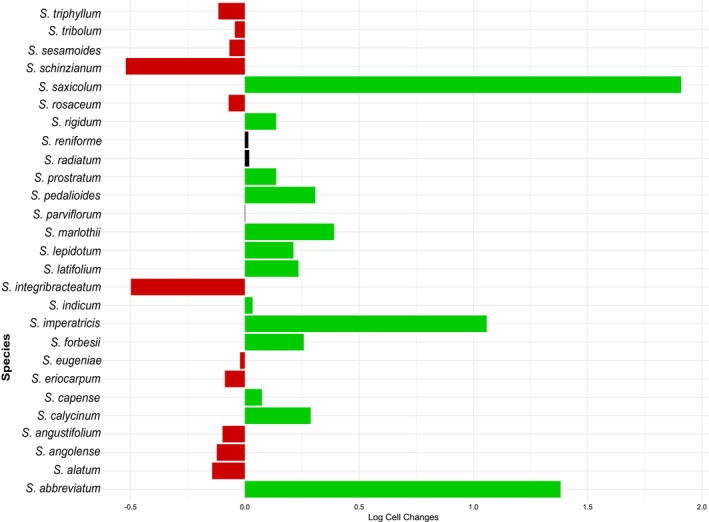
Niche breadth sizes and habitat suitability of all *Sesamum* species based on the present and future predictions.

According to SDMs, *Sesamum* species exhibited contrasting niche breadth responses to environmental variables, as seen in the comparisons of predicted niche breadth sizes between the current and future (2080) scenarios (Figures 4 and 5). Comparing species, niche breadths from the present to 2080, approximately 46.42% (13 species) are expected to experience a reduction in their niche breadths. *Sesamum schinzianum* (Figure 4A) experiences the most significant decline case scenario with a −40.60% decrease, suggesting a substantial contraction in its geographical range. Other species, like *S. sesamoides* (Figure 4C), although widely distributed from west to central into east Africa and to some part of subtropical southern Africa, will experience a range reduction of about −6.52% (Figure 4). *Sesamum integribracteatum* (−39.07%) also shows considerable reductions, indicating these species might face significant habitat loss or become more restricted in their distribution. About 39.29% (11 species) are predicted to increase their range sizes in the future. For example, *S. abbreviatum* (Figure 4B) and the eastern African *S. saxicola* and *S. forbesii* (Figure 4C) show the highest increase in range size with 188.15% and 298.1% rises, respectively. While 12% (4 species) are predicted to maintain or undergo negligible change in their niche breadths. For example, the southern African species *S. parviflorum* (Figure 4E) and *S. eriocarpum* (Figure 4F) have negligible changes of 0.16% and 0.15%, respectively, indicating stable geographical distributions (Figures 4 and 5).

### 3.4 Phylogenetic Signal

**FIGURE 6:**
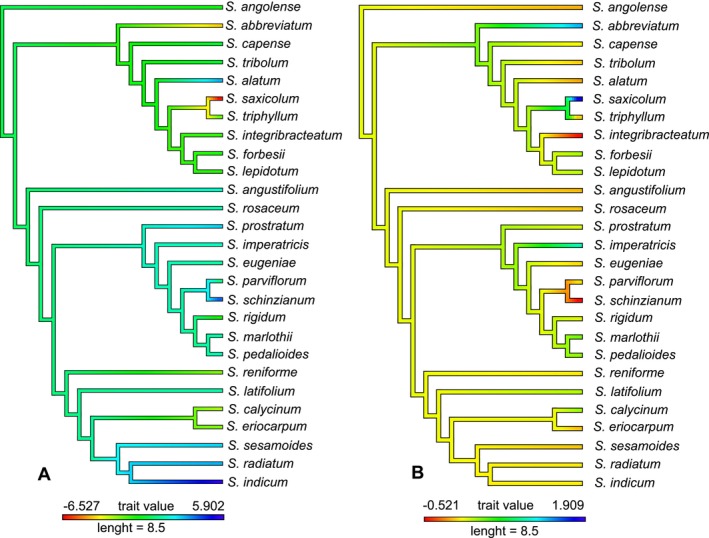
Phylogenetic signal for (A) niche breadth and (B) range size, in *Sesamum* species.

Mapping species niche breadths and extinction risk onto the *Sesamum* phylogenetic tree indicated a random phylogenetic structure, as confirmed by Blomberg's K and Pagel's λ tests (Figure 6).

## 4 Discussion

### 4.1 Model Validation

The high AUC values for predicting suitable habitats for *Sesamum* species align with previous studies that have highlighted the utility of the Maxent model in ecological niche modeling and species distribution predictions (Elith et al. 2011; Lorestani et al. 2022; Tesfamariam et al. 2022; Valavi et al. 2022; Ahmadi et al. 2023). Key environmental variables were identified as significant contributors to the habitat suitability for the studied species. For example, Bio13 and Bio14 are crucial factors for many species, highlighting the critical role of precipitation patterns. This finding is consistent with other research that emphasizes the importance of precipitation in shaping species distributions, particularly in regions where water availability is a limiting factor (e.g., Hijmans et al. 2005; Kumar et al. 2009; Yao et al. 2022). Temperature extremes also play a significant role in determining habitat suitability, as evidenced by the high contribution of Bio5 for species such as *S. angolense* and *S. calycinum* . This suggests that these species are particularly sensitive to maximum temperature variations, which could be a crucial factor in their ecological niche. Similar observations have been made in studies of other taxa, where temperature extremes were found to be critical determinants of species distributions (Wiens et al. 2009; Oliveira et al. 2022). Bio19 and bio2 were also important for certain species, indicating that both temperature and precipitation play vital roles in habitat suitability. However, the specific impact of these variables varies among species, suggesting that each species has unique ecological requirements. This variability in environmental variable contributions underscores the complexity of ecological niches and the need to consider multiple factors when modeling species distributions (Franklin 2010; Peterson et al. 2011). Despite a general trend in the influence of climatic variables on species distributions, the study also highlights the significant variation in environmental variable contributions among the different species. This variation suggests that while climatic factors are crucial, edaphic factors also play important roles in shaping species distributions. Each species responds differently to these factors, reflecting their unique ecological niches and adaptive strategies (Soberón and Peterson 2005). These findings underscore the importance of considering a range of environmental variables in species distribution models and highlight the complex interplay between climatic and edaphic factors in determining habitat suitability. The high predictive performance of the Maxent model, combined with its ability to identify key environmental determinants, makes it a valuable tool for ecological niche modeling and conservation planning.

### 4.2 SDM Implications

These findings highlight the significance of PC1 in predicting the distribution of Sesamum species, where higher PC1 values are associated with more suitable niche breadths, suggesting that environmental factors represented by PC1 play a crucial role in shaping the distribution of Sesamum species. The PCA analysis revealed that PC1 effectively captures variation in niche breadth among *Sesamum* species, accounting for 52.4% of the total variance. Species with higher PC1 scores, such as *S. indicum* and *S. schinzianum*, exhibit broader niches and larger geographic ranges, aligning with the concept of niche breadth as defined by MacArthur (1972) and Hausharter et al. (2023). Conversely, species with lower PC1 scores, like *S. saxicola* and *S. abbreviatum* , have narrower niches and more restricted distributions. These findings suggest that the ability to tolerate a wider range of environmental conditions is associated with increased geographic range size, consistent with previous studies (cite relevant studies). Mean diurnal range temperature and precipitation positively contributed to PC1, indicating that species adapted to varying thermal and hydrological regimes tend to have broader niches. In contrast, soil type negatively influenced PC1, suggesting that species with specific soil requirements, such as *S. trifolium* , exhibit narrower niches (Table 1). The regression analysis confirms that PC1, as an index of niche breadth, is a significant predictor of range size in *Sesamum* species. Species with broader niches, as indicated by higher PC1 scores, are predicted to have larger geographic ranges. These findings are consistent with the ecological theory that generalists with wider niche tolerances tend to have larger distributions compared to specialists with narrower niches (MacArthur 1972; Zhang et al. 2023). This analysis strengthens the evidence that PC1 can be a valuable tool for understanding the ecological relationships between *Sesamum* species and their environment.

### 4.3 Quantification of Range Extent

The SDM for *Sesamum* species provides critical insights into their current and potential future distribution patterns, particularly in response to climate change. The analysis of niche breadths under present and projected future (2080) scenarios reveals significant variability in how different species will respond to environmental changes. Approximately 39% of the *Sesamum* species are projected to experience reductions in their niche breadths by 2080. This trend is alarming, as it suggests that most species will face increasingly restrictive habitat conditions, potentially leading to range contractions. Notably, *S. schinzianum* is expected to undergo the most severe reductions in niche breadth. These findings align with broader ecological research indicating that climate change can lead to habitat loss and reduced distributional ranges for many plant species (Bellard et al. 2012; Urban 2015; Auffret and Svenning 2022; Weiskopf et al. 2024).

Conversely, about 46% of the species, including the southern African *S. integribracteanum* and *S. marlothii*, are predicted to expand their niche breadths. This suggests that some *Sesamum* species may benefit from changing climatic conditions, potentially finding new suitable habitats or expanding into previously uninhabitable areas. This phenomenon has been observed in other plant species as well, where shifts in climate can create new opportunities for colonization and growth (e.g., Parmesan 2006; Zu et al. 2021; Moran et al. 2022; Zhigila et al. 2023). The remaining 12% of the *Sesamum* species, such as *S. parviflorum* (Figure 4E) are expected to maintain their current niche breadths or experience negligible changes. These species appear to be resilient to climatic fluctuations, possibly due to their broad ecological tolerance or adaptive traits that buffer against environmental variability. Studies have shown that species with stable niches often possess traits that allow them to thrive under a wide range of environmental conditions (Thuiller et al. 2005; Wiens et al. 2010).

The contrasting responses of *Sesamum* species to future climate scenarios underscore the importance of targeted conservation strategies. Species predicted to experience significant niche reductions, such as *S. schinzianum*, may require immediate conservation interventions, such as habitat protection and restoration efforts, to prevent potential declines or extinctions. On the other hand, species projected to expand their niches might become more widespread, but this could also lead to competitive interactions with native species in newly colonized areas (Early and Sax 2014).

### 4.4 Range Size and Extinction Risk Are Not Phylogenetically Conserved

Our study found that niche breadth and extinction risk of *Sesamum* species exhibited a very weak phylogenetic signal. This aligns with the previous findings that species range size is not phylogenetically structured but is instead influenced significantly by both external spatiotemporal environmental factors and the intrinsic traits of the species (Estrada et al. 2015). Further, studies have demonstrated that range size is more indicative of a species' spatial tracking of its environmental niches in response to changing conditions, such as temperature variations (Rubenstein et al. 2023; Polo et al. 2024).

Although this study provides insights into the vulnerability of *Sesamum* species to climate change, it has some limitations that warrant further investigation. The reliance on MaxEnt models and static environmental variables may oversimplify ecological dynamics, potentially overlooking biotic interactions, microhabitat variability, and dispersal constraints. Additionally, the inherent uncertainty in future climate projections and the lack of extensive field validation limit the predictive accuracy of range shifts (Couet et al. 2022). To address these gaps, future research should integrate dynamic climate models, biotic interactions, and trait‐based approaches, alongside comprehensive field surveys and genomic studies. These advancements will refine predictions, enhance conservation prioritization, and provide a more robust understanding of *Sesamum* species' adaptive capacities and extinction risks.

### 4.5 Conclusions

Our findings highlight the vulnerability of *Sesamum* species to climate change. While species with broader niches currently occupy larger ranges, the projected range contractions are driven by key climatic variables such as precipitation of the wettest month, temperature fluctuations, and mean cold‐season temperatures, as well as soil characteristics such as pH and clay content. Species with narrow ecological niches, for example, *S. saxicola* and *S. abbreviatum* , are especially at risk in regions facing rising temperatures and prolonged droughts, while areas with stable climates may serve as future refugia. The interplay between climatic and edaphic factors shaping *Sesamum* species distributions emphasizes the importance of comprehensive conservation strategies. Prioritizing species with significant range contractions is essential to prevent population declines and potential extinction. Understanding the specific ecological requirements of each species will be crucial for developing targeted conservation actions, including habitat restoration, assisted migration, ex situ conservation, and climate‐adaptive agricultural practices. Long‐term monitoring of *Sesamum* populations and continued research on climate change impacts are necessary to inform adaptive management strategies and ensure the persistence of these valuable plant species.

## Author Contributions

**Daniel A. Zhigila:** conceptualization (equal), data curation (equal), formal analysis (equal), funding acquisition (equal), investigation (equal), methodology (equal), resources (equal), validation (equal), visualization (equal), writing – original draft (equal), writing – review and editing (equal). **Nawal Shrestha:** formal analysis (equal), validation (equal), visualization (equal), writing – review and editing (equal). **Zainab A. Abubakar:** project administration (equal), supervision (equal), validation (equal), visualization (equal), writing – review and editing (equal). **A. Muthama Muasya:** conceptualization (equal), data curation (equal), funding acquisition (equal), investigation (equal), methodology (equal), project administration (equal), resources (equal), supervision (equal), validation (equal), visualization (equal), writing – review and editing (equal).

## Conflicts of Interest

The authors declare no conflicts of interest.

## Supporting information


**Table S1.** Geographic coordinates (longitude and latitude) of occurrence records for *Sesamum* species used in this study. These data were used for species distribution modeling and spatial analyses.

## Data Availability

The data that support the findings of this study are openly available in GenBank at https://www.ncbi.nlm.nih.gov/Genbank/update.html, GenBank accession numbers OM746317–OM746339.
